# Population genetic diversity and structure of *Rhinogobius candidianus* (Gobiidae) in Taiwan: Translocation and release

**DOI:** 10.1002/ece3.9154

**Published:** 2022-08-11

**Authors:** Bin Kang, Kui‐Ching Hsu, Jui‐Hsien Wu, Yuh‐Wen Chiu, Hung‐Du Lin, Yu‐Min Ju

**Affiliations:** ^1^ The Key Laboratory of Mariculture (Ocean University of China) Ministry of Education Qingdao China; ^2^ College of Fisheries Guangdong Ocean University Zhanjiang China; ^3^ Eastern Marine Biology Research Center of Fisheries Research Institute Council of Agriculture Taitung Taiwan; ^4^ Department of Biological Resources National Chiayi University Chiayi Taiwan; ^5^ The Affiliated School of National Tainan First Senior High School Tainan Taiwan; ^6^ National Museum of Marine Biology and Aquarium Pingtung Taiwan; ^7^ Department of Marine Biotechnology and Resources National Sun Yat‐sen University Kaohsiung Taiwan

**Keywords:** approximate Bayesian computation, demography, mitochondria, phylogeography, *Rhinogobius candidianus*, translocated

## Abstract

*Rhinogobius candidianus* is a freshwater goby distributed in north, northwest, west, and south Taiwan, but this species has been introduced to east Taiwan and became dominant. To investigate its native population genetic diversity and structure and evaluate the sources and diversity of translocated populations, the mitochondrial DNA control region and cytochrome *b* gene (1981 bp) from 220 specimens were analyzed. These results indicated that (1) the east populations originated from two sources in west Taiwan; (2) translocated populations exist in east Taiwan and south Taiwan; (3) many populations have likely been moved secondarily by human intervention; (4) the effective size of the populations had declined greatly; (5) within the native populations, the ancestral populations colonized Taiwan during the land bridge phase in the Pleistocene through north Taiwan; (6) the landform changes in Taiwan shaped the population structure; and (7) the landforms of the coastline during glaciation also shaped the native range. The low‐level genetic diversity, high population differentiation, and population decline greatly suggest the need for resource management and conservation interventions. Four clades (*α*–*δ*) should be managed as four distinct evolutionarily significant units, while the translocated populations should be managed as separate management units. Moreover, the translocated populations in east Taiwan should be evaluated and monitored carefully.

## Graphical abstract

The phylogeography of Rhinogobius candidianus in Taiwan Island is uncertain. This study provides the evidence to confirm the populations in east Taiwan and Miaoli Plateau as translocation populations, and some specimens in native populations from secondarily moved by human intervention.

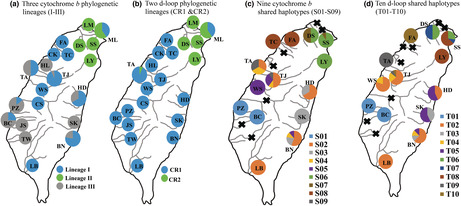

## 1 INTRODUCTION

*Rhinogobius* is a genus of freshwater goby native to East Asia. Most species are primarily freshwater fishes, and very few species are amphidromous. In the Catalog of Fishes (Fricke et al., 2019), there are currently 83 valid species listed within this genus. In FishBase (Froese & Pauly, 2022), there are 108 scientific names, but only 65 are valid species. Currently, new *Rhinogobius* species are being identified (Takahashi & Okazaki, 2017; Xia et al., 2018), and these new species descriptions may suggest the list is not yet complete.

Of the 10 described *Rhinogobius* species on Taiwan island, five species have been analyzed for their phylogeographic relationships; *R. maculafasciatus* (Cheng et al., 2005a), *R. rubromaculatus* (Cheng et al., 2005b), *R. giurinus* (Ju et al., 2016), *R. delicatus* (Ju et al., 2021), and *R. gigas* (Liao et al., 2021). Recently, our study found that the *Rhinogobius* species have been exported for the worldwide aquarium trade from Taiwan (our observations; Yang et al., 2020). Besides *R. giurinus*, an amphidromous species, the distribution area of *R. candidianus* is wider than that of other Taiwan species. Chen and Shao (1996) proposed that *R. candidianus* was distributed in north, northwest, west, and south (north of Tzengwen River) Taiwan, but Leander et al. (2014) found that this species was also distributed in east Taiwan, and in and south of the Tzengwen River. Liao et al. (2021) proposed that *R. candidianus* has been introduced to east Taiwan and became dominant. Thus, our study analyzed mitochondrial DNA in order to characterize the genetic diversity of *R. candidianus* and determine the geographic origin of translocated stocks as well as any human‐mediated gene flow between translocated stocks and native populations.

**FIGURE 1:**
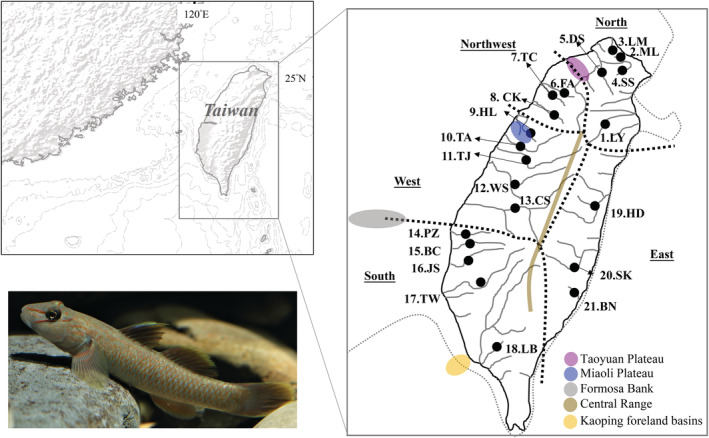
Sampling localities of the *Rhinogobius candidianus* in Taiwan. Black dots represent approximate sampling sites. Sample size is given in Table 1. The black thicker dashed lines represent the border of each zoogeographical district, which were defined by Tzeng (1986). The gray thinner dotted lines represent the coastline during the glaciations following Ota (1998). The organism photograph was provided by Mingtai Zhou.

Previous studies (Chang et al., 2016; Chiang et al., 2010, 2013; Chiu et al., 2017; Han et al., 2019) have suggested that Taiwan island provides an excellent opportunity for examining phylogeographic patterns. Taiwan island is located off the southeastern coast of mainland China and the southern East Asian islands. Geological evidence indicates that land bridges connected this island to the Asian continent and the Japanese islands during glaciations (Fairbanks, 1989; Gascoyne et al., 1979; Kimura, 2000; Ota, 1998). Past phylogeographic studies have highlighted the influence of natural geological barriers, and general heterogeneous topography of Taiwan, in shaping the regional biogeography of freshwater fishes (Chiang et al., 2010, 2013; Chiu et al., 2017; Han et al., 2019; Ju et al., 2018; Lin et al., 2016). Many ichthyofaunal and phylogeographic studies have suggested that geological barriers such as the Central Range, Miaoli Plateau, and Kaoping foreland basins shaped the structures and distribution patterns (Han et al., 2019; Ju et al., 2018; Lin et al., 2016) (Figure 1). Thus, the distribution patterns of freshwater fishes in Taiwan vary. For example, *Opsariichthys pachycephalus*, *Candidia barbatus*, *Cobitis sinensis*, and *Acrossocheilus paradoxus* are widely distributed in north, west, and south Taiwan; *Sinogastromyzon puliensis* is distributed south of the Miaoli Plateau; and *O. evolans*, *Squalidus argentatus*, *Sinibrama macrops*, and *Hemibarbus labeo* are only distributed in the Tamsui River, north of the Taoyuan Plateau. Lin et al. (2016) proposed that the different distribution patterns of freshwater fishes in Taiwan resulted from different colonization times and colonization routes contributed by the geological history. Thus, our study attempted to examine the intraspecific genetic diversity of native *R. candidianus* to understand its native distribution pattern, structure, diversity, and colonization history.

Accordingly, the present major aims were to determine (1) the native, translocated, and released populations of *R. candidianus*, (2) the demography and genetic diversity of native *R. candidianus*, and (3) the genetic structure and colonization history of native *R. candidianus* in Taiwan. To achieve the above aims, the genetic variability was analyzed using mitochondrial DNA (mtDNA) cytochrome *b* gene (cyt *b*), and control region (CR or d‐loop). The regions of the mtDNA are often analyzed in studies of animal phylogeography (e.g., Chen et al., 2007; Hsu et al., 2014; Yang et al., 2016). Moreover, these two loci have previously been used in the analysis of Taiwanese freshwater fishes and freshwater gobies (Chiang et al., 2010, 2017; Ju et al., 2018, 2021; Liao et al., 2021).

## 2 MATERIALS AND METHODS

The care and use of experimental animals complied with Taiwan's animal welfare laws, the Taiwan Council guidelines and policies as approved by MOST 106‐2611‐M‐291‐007. Taiwan's government does not require any license to work with dead animals from fisheries. All methods and the handling of fish were performed and approved by the National Museum of Marine Biology and Aquarium.

### 2.1 Population sampling and molecular methods

A total of 220 specimens of *R. candidianus* were collected from 21 localities across Taiwan (Table 1, Figure 1). All specimens are housed in the laboratory of Yu‐Min Ju, National Museum of Marine Biology and Aquarium. These specimens were classified into five groups, north, northwest, west, south, and east, based on previous ichthyofaunal and phylogeographic studies (e.g., Han et al., 2019; Tzeng, 1986; Wang et al., 2004) (Table 1, Figure 1). Five sampling localities belong to north Taiwan: (1) Lanyang, LY; (2) Malain, ML; (3) Laomei, LM; (4) Shuangxi, SS; and (5) Danshuei, DS. Three localities belong to northwest Taiwan: (6) Fongshan, FA; (7) Touqian, TC; and (8) Chongkang, CK. Five localities belong to west Taiwan: (9) Houlong, HL; (10) Taan, TA; (11) Tajia, TJ; (12) Wuxi, WS; and (13) Chuoshuei, CS. Five localities belong to south Taiwan: (14) Puzih, PZ; (15) Bachang, BC; (16) Jishui, JS; (17) Tzengwen, TW; and (18) Liben, LB. Three sampling localities belong to east Taiwan: (19) Hualian, HD; (20) Siukuluan, SK; and (21) Beinan, BN. Fish were collected from field sites with seines and fatally anesthetized with MS‐222 (Sigma). The samples were fixed and stored in 100% ethanol. Genomic DNA was extracted from muscle tissue using a genomic DNA purification kit (Gentra Systems, Valencia, CA). The entire cyt *b* gene and CR region were amplified by polymerase chain reaction (PCR) using primers from Xiao et al. (2001) and Zhou et al. (2017). Each 50 μl PCR reaction mixture contained 5 ng of template DNA, 5 μl of 10x reaction buffer, 4 μl of dNTP mix (10 mM), 5 pmol of each primer, and 2 U of Taq polymerase (TaKaRa, Taq polymerase). The PCR was programmed on an MJ Thermal Cycler as 1 cycle of denaturation at 94°C for 4 min, 40 cycles of denaturation at 94°C for 30 s, annealing at 51°C–55°C for 50 s–1 min, and extension at 72°C for 1 min 30 s, followed by a 72°C extension for 10 min and 4°C for storage. The purified PCR products were sequenced using an ABI 377 automated sequencer (Applied Biosystems, Foster City, CA, USA). The chromatograms were assessed using the software CHROMAS (Technelysium), and the sequences were manually edited using BioEdit 6.0.7 (Hall, 1999).

**TABLE 1:** Samples used for analysis, location, code, and summary statistics, including private haplotype (Hp), shared haplotype within population (Hw), haplotype diversity (h), and nucleotide diversity (*θ*_π_)

Region	Basin	Code	*N*	Cyt *b*					D‐loop				
				allele	Hp	Hw	*h*	*θ*_π_	allele	Hp	Hw	*h*	*θ*_π_
North	Lanyang	1.LY	10	3	2	2	0.644	0.003	3	2	2	0.511	0.001
	Malain	2.ML	10	2	2	2	0.533	0.009	5	5	2	0.756	0.007
	Laomei	3.LM	9	3	3	3	0.667	0.006	4	4	2	0.694	0.002
	Shuangxi	4.SS	11	2	0	0	0.182	0.000	3	0	0	0.564	0.001
	Danshuei	5.DS	13	10	8	2	0.949	0.003	10	8	2	0.949	0.009
Northwest	Fongshan	6.FA	9	1	0	0	NA	NA	5	4	3	0.806	0.005
	Touqian	7.TC	9	2	1	0	0.222	0.001	5	5	2	0.806	0.003
	Chongkang	8.CK	10	3	3	2	0.511	0.002	3	3	2	0.511	0.003
West	Houlong	9.HL	22	4	4	2	0.455	0.001	6	6	4	0.779	0.003
	Taan	10.TA	14	11	8	3	0.967	0.012	8	7	2	0.923	0007
	Tajia	11.TJ	14	5	1	1	0.791	0.005	4	0	0	0.571	0.002
	Wuxi	12.WS	10	1	0	0	NA	NA	2	0	0	0.356	0.001
	Chuoshuei	13.CS	10	2	2	2	0.556	0.001	4	4	2	0.711	0.002
South	Puzih	14.PZ	7	4	3	2	0.810	0.004	5	4	1	0.857	0.006
	Bachang	15.BC	8	2	1	1	0.429	0.008	4	3	2	0.821	0.005
	Jishui	16.JS	11	1	1	1	NA	NA	3	3	3	0.691	0.002
	Tzengwen	17.TW	11	4	4	3	0.745	0.003	6	6	2	0.855	0.003
	Linben	18.LB	4	1	0	0	NA	NA	1	0	0	NA	NA
East	Hualian	19.HD	9	4	2	1	0.778	0.015	4	2	0	0.750	0.009
	Siukuluan	20.SK	10	2	1	1	0.467	0.000	3	1	1	0.733	0.001
	Beinan	21.BN	9	4	0	0	0.694	0.011	5	1	0	0.722	0.007
	Total		220	55			0.965	0.016	78			0.971	0.012

### 2.2 Sequence alignment and phylogenetic inferences

The nucleotide sequences were aligned with Clustal X 1.81 (Thompson et al., 1997). The most appropriate nucleotide substitution model was TrN + I + G using the Bayesian information criterion (BIC) for cyt *b*, CR, and concatenated data sets in jmodelTest 2.0 (Darriba et al., 2012). The phylogenetic analysis was performed using a maximum likelihood (ML) estimation with MEGA‐X (Kumar et al., 2018). Bootstrapping was performed with 1000 replications. The median‐joining algorithm (Bandelt et al., 1999) from Network 5.0 was used to reconstruct the haplotype networks. Additionally, the phylogenetic tree was also generated by the program BEAST 1.8.0 (Drummond et al., 2013) with 10^7^ MCMC steps and the first 10% as burn‐in. The strict clock model with a Bayesian skyline tree was used to construct Bayesian skyline plots and estimate the divergence times of the major lineages to the most recent common ancestor (*T*_MRCA_) by running 10^6^ generations. The molecular clock was calibrated using a divergence rate of 1.33% per million years for the concatenated data set (Burridge et al., 2008; Ju et al., 2021). The output was visualized in Tracer v1.6 (Rambaut et al., 2013) to determine that convergence and suitable effective sample size were achieved for all parameters. The burn‐in and plots for each analysis were visualized using Tracer. The TreeAnnotator in the BEAST package was used to summarize the tree data, and the tree was viewed using FigTree v1.3 (Rambaut, 2014).

### 2.3 Population diversity and structure

The levels of intrapopulation genetic diversity were estimated based on indices of haplotype diversity (*h*) (Nei & Tajima, 1983) and nucleotide diversity (θ_π_) (Jukes & Cantor, 1969) in DnaSP 4.10.8 (Rozas et al., 2003). Two genetic differentiation indices, *G*_ST_ and *N*_ST_, were used to examine the existence of a phylogeographic structure in DnaSP (Pons & Petit, 1996). Pairwise *F*_ST_ and *p*‐distance values implemented by DnaSP were used to examine the spatial partitioning of genetic variation among populations. To determine the possible diversification scenarios, a statistical dispersal‐vicariance analysis (S‐DIVA) was employed to determine statistical support for ancestral range reconstructions (Yu et al., 2010). The S‐DIVA analysis was implemented in RASP v.3.1 (Yu et al., 2015). Range information was defined using the ichthyofaunal classification (Tzeng, 1986) (Table 1, Figure 1). The analysis was performed using the ‘maxareas = 4–7’ option.

### 2.4 Population demography and history

The past demographic expansions were examined using three different approaches. First, Tajima's *D* (Tajima, 1989) and Fu's *Fs* (Fu, 1997) tests were used in DnaSP. Second, the demographic history was examined using mismatch distribution analyses implemented in DnaSP. Thirdly, the effective population size changes over time were evaluated using the Bayesian Skyline Plots (BSP) computed with BEAST. A Bayesian Skyline tree was selected and a strict clock model was used. We ran the analysis for 3 × 10^7^ generations to ensure convergence of all parameters (ESSs > 200) and the first 10% of samples for each chain were discarded as burn‐in. We used the same setting for two independent runs to check for convergence with 3 × 10^7^ and sampling every 1 × 10^3^ generations. Plots for each analysis were drawn using Tracer.

The Approximate Bayesian Computation (ABC) framework was used to determine the population history with the software DIYABC ver. 2.0 (Cornuet et al., 2014). The reference table was built with 1,000,000 simulated data sets per scenario using all statistics. We used uniform priors for all scenarios and gave no constraints to population sizes and coalescent times. The posterior probabilities were compared by logistic regression. To examine the colonization history, our study tested four simple population scenarios. The scenarios were as follows: In scenario 1 (South model), the populations colonized in south Taiwan, and then distributed northward widely. In scenario 2 (Northwest model), the populations colonized in northwest Taiwan, and then distributed northward and southward. In scenario 3 (West model), the populations colonized in west Taiwan, and then distributed northward and southward. In scenario 4 (North model), the populations colonized in north Taiwan, and then distributed southward.

## 3 RESULTS

### 3.1 Native, translocated, and released populations

**FIGURE 2:**
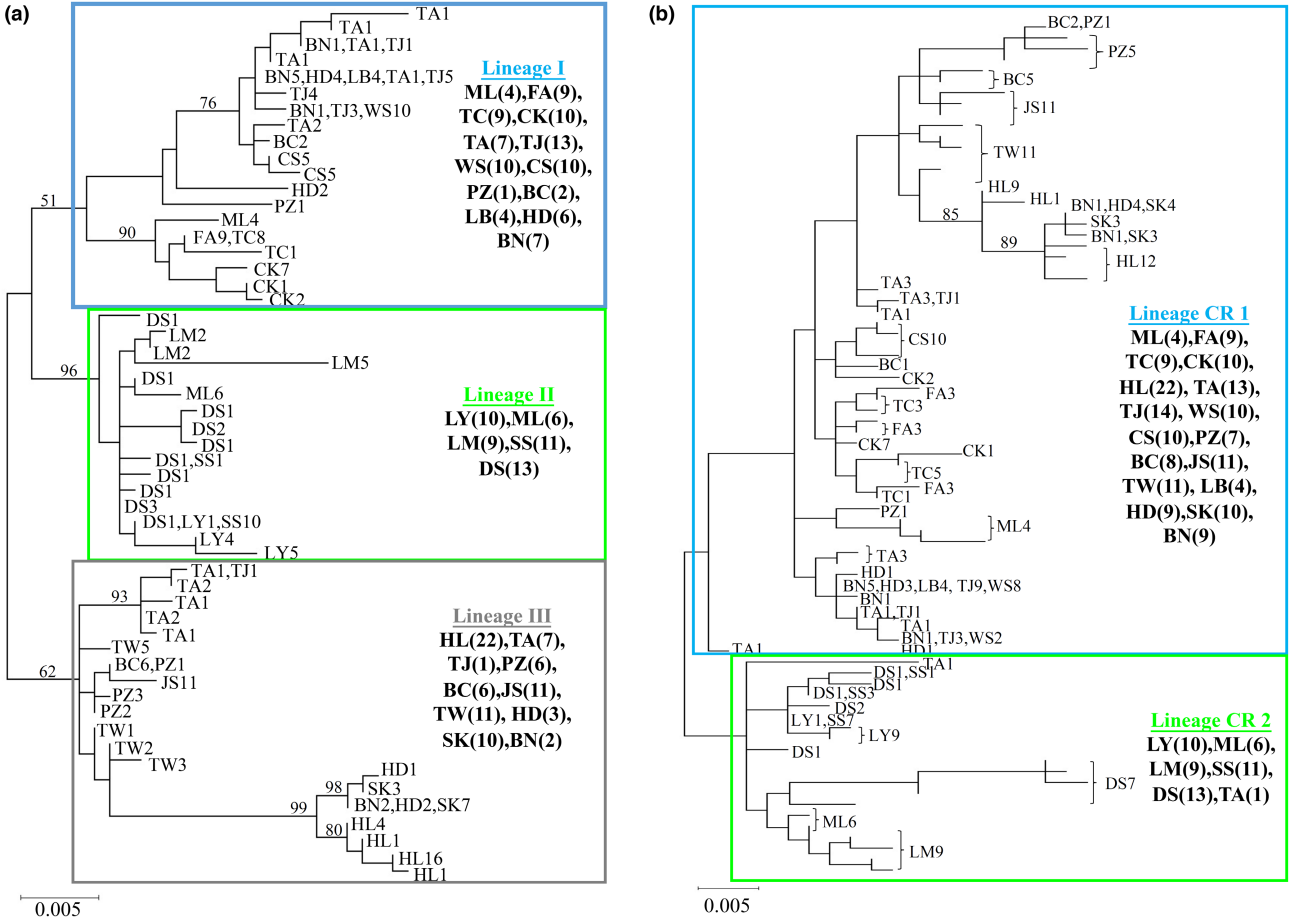
The maximum likelihood (ML) tree for *Rhinogobius candidianus*. Based on (a) 55 haplotypes of cytochrome b sequences (1141 bps) and (b) 78 haplotypes of d‐loop sequences (840 bps). The numbers at the nodes are bootstrap values.

**FIGURE 3:**
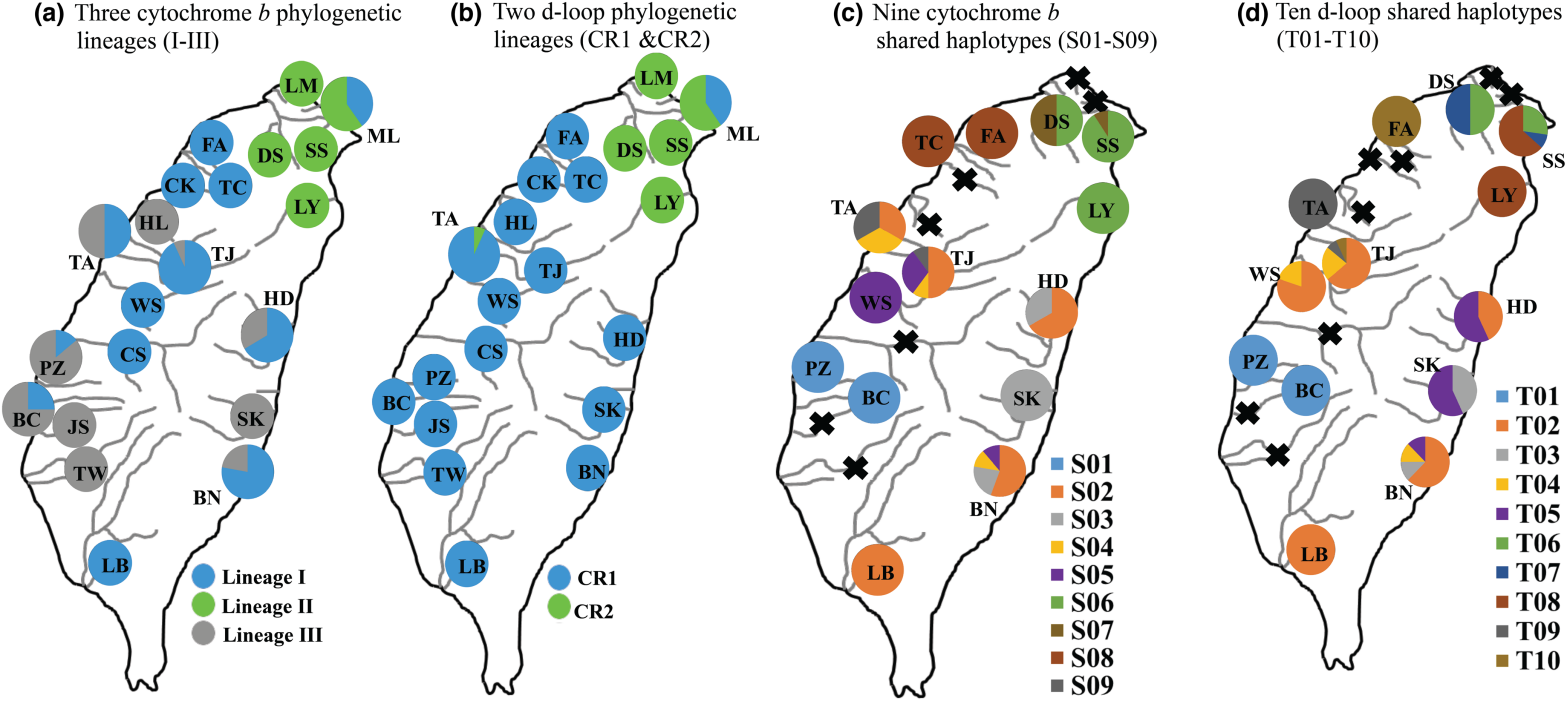
The distribution of (a) the cytochrome b phylogenetic lineages (I–III) in Figure 2a, (b) the d‐loop phylogenetic lineages (CR1 & CR2) in Figure 2b, (c) the shared cytochrome b haplotypes (S01‐S09), and (d) shared d‐loop haplotypes (T01‐T10). Cross represents no shared haplotype.

A total of 1981 base pairs (bp) of mtDNA sequences [1141 bp of the cyt *b* (104 variable sites and 89 phylogenetically informative sites) and 840 bp of the CR (66 variable sites and 55 phylogenetically informative sites)] from 220 specimens were analyzed. In the cyt *b* data set, a total of 55 haplotypes were obtained (refer to Table 1). The ML phylogenetic tree revealed that these 55 haplotypes fall into three lineages (lineages I, II, and III) (Figure 2a). The Bayesian tree revealed the same topology (data not shown). These three lineages were sympatric in some populations (Figure 3a). Among the 55 haplotypes, only nine were shared by two or more populations, and six shared haplotypes (S01, S03, S06, S07, S08, and S09) were only distributed in adjacent populations (Figure 3c). The S02, S04, and S05 were distributed in the west and east populations. Five populations (SS, FA, WS, LB, and BN) did not have private haplotypes (Table 1). Four populations (FA, WS, JS, and LB) only included one haplotype.

In the CR data set, a total of 78 haplotypes were obtained (refer to Table 1). The ML phylogenetic tree revealed that these haplotypes fall into two major lineages (CR1 and CR2), but not all clusters were highly supported by bootstrapping test (Figure 2b). The Bayesian tree revealed similar topology (data not shown). These two lineages were only sympatric in populations ML and TA (Figure 3b). Among the 78 haplotypes, only 10 were shared by two or more populations, and eight shared haplotypes (T01, T03, T05, T06, T07, T08, T09, and T10) were only distributed in adjacent populations (Figure 3d). The T02 and T04 were distributed in the west and east populations. Four populations (SS, TJ, WS, and LB) did not have private haplotypes (Table 1). The population LB included only one haplotype (Table 1).

Based on the results obtained from the cyt *b* and CR data sets (Table 1, Figures 2 and 3), we assumed that the population LB originated from human‐mediated movement because this population did not have private haplotypes, all specimens revealed the same haplotype in cyt *b* and CR data sets, and only this one haplotype (S02 in cyt *b* and T02 in CR; Figure 3c,d) was shared between other populations (BN, HD, TA, TJ, and WS). Most shared cyt *b* and CR haplotypes were only distributed in adjacent populations (Figure 3c,d), but the east and west populations shared the same haplotypes [e.g., populations HD and BN (east) and populations TA and TJ (west) all included S02 and T02; and population BN (east) and population TJ (west) both included S02, S04, S05, T02, and T04]. Thus, our study suggested that the four populations LB, HD, SK, and BN originated from human intervention and these populations were not distributed in the native area, and thus are defined as translocated populations (see 4.2 Native, translocated, and released populations). In addition, population SK and the other two east populations, HD and BN, shared haplotypes S03, T03, and T05, and these three haplotypes were only distributed in these east populations. This suggests that these three populations may have recently diverged from common sources of the east populations.

**FIGURE 4:**
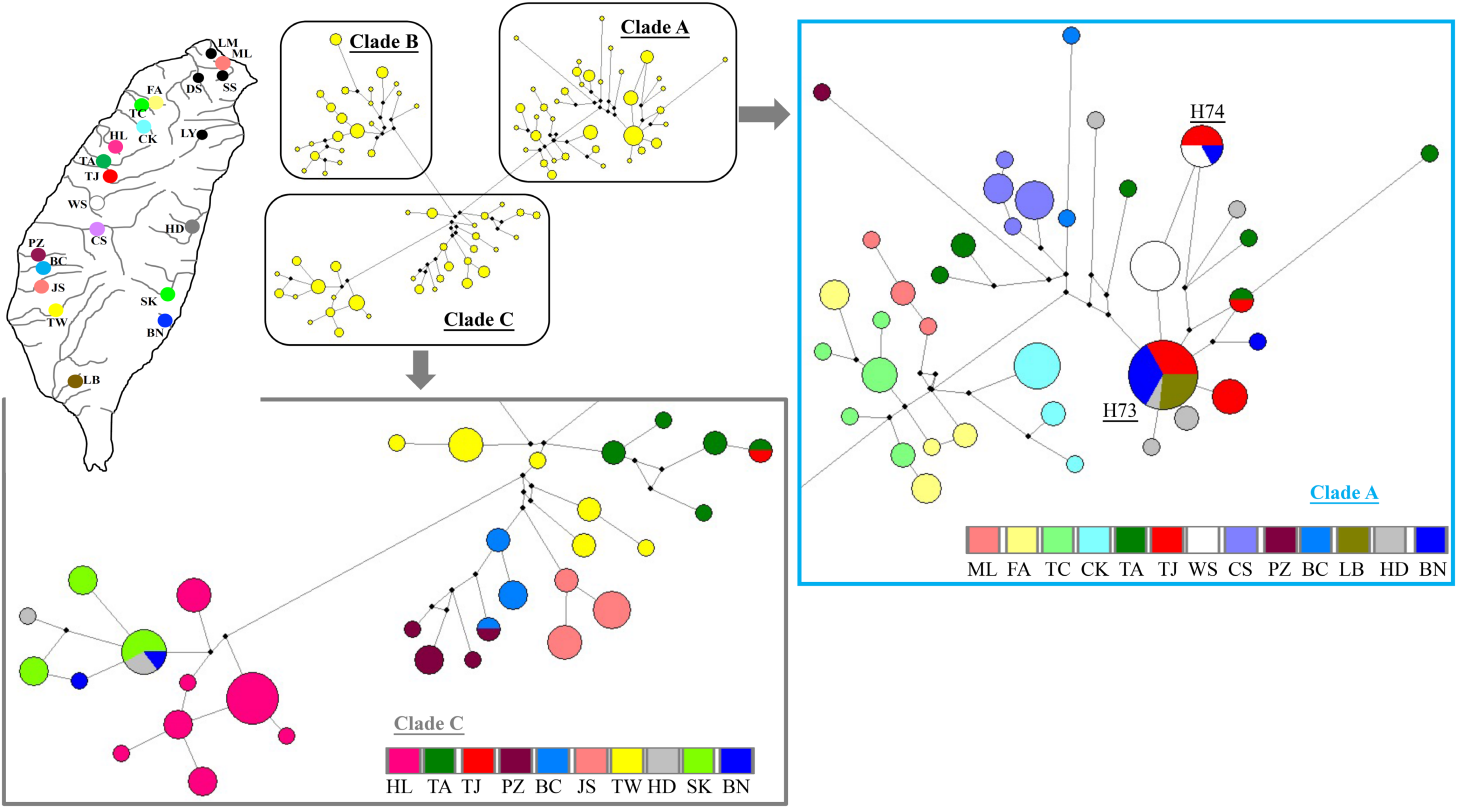
The median‐joining network of 91 concatenated mtDNA sequences (1981 bps) of all *Rhinogobius candidianus*

The cyt *b* lineages I and III displayed disjunct distributions (Figure 3a), but these populations were almost all grouped together as lineage CR1 based on the d‐loop region (Figure 3b). To identify the relationships among these populations, our study investigated genetic diversity and population structure further using the concatenated data set. In the mtDNA haplotype network, all 91 mt haplotypes were assorted as three clades (Figure 4). This pattern was the same as the pattern of cyt *b* data set (Figures 2a and 3a). The three concatenated clades (clades A–C) corresponded to the three cyt *b* lineages (lineages I–III). The network revealed that these four presumed translocated populations (LB, HD, SK, and BN) might originate from two sources. Within clade A, some specimens in populations LB, HD, and BN might originate from populations TJ. Within clade C, some specimens in populations HD, SK, and BN might originate from population HL. However, these four presumed translocated populations are far from populations TJ and HL in geography (Figures 1 and 4).

Within clade C, the populations HL, TA, and TJ were not close to population TW in geography, but the haplotype network grouped these three populations close to population TW (Figures 1 and 4). Within clade A, the population ML was not close to populations FA, TC, and CK in geography, but the haplotype network grouped ML with specimens from populations FA, TC, and CK (Figures 1 and 4). The pairwise p‐distance values among populations also supported these results within each of the clades (Table 2). Although disjunct distributions could be the result of extinctions, our study considered that these specimens in population ML within clade A, and these specimens in populations HL, TA, and TJ within clade C were secondarily moved through human intervention. Moreover, within clade A, the results of the pairwise p‐distance revealed that the populations PZ and BC were close to the populations LB, HD, and BN (Table 2), although the haplotype network showed that they might originate from population CS (Figure 4). The population CS only included the cytochrome *b* phylogenetic lineage I, but the populations PZ and BC included lineages I and III (Figures 2a and 3a). Thus, our study also suggests that some specimens in populations PZ and BC originated secondarily through human movement. Because the populations were distributed in the original native area (see 4.2 Native, translocated, and released populations), our study defined those specimens in populations ML, HL, TA, TJ, PZ, and BC as released populations (Table 3).

**TABLE 2:** Matrix of *p*‐distance (*10^−2^; below) with concatenated clades A, B, and C in Figure 4

**Clade A**													
	ML	FA	TC	CK	TA	TJ	WS	CS	PZ	BC	LB	HD	BN
ML													
FA	0.8												
TC	0.7	0.2											
CK	0.9	0.5	0.5										
TA	1.3	1.1	1.1	1.1									
TJ	1.2	0.9	0.9	1.0	0.4								
WS	1.1	0.9	0.9	1.1	0.5	0.2							
CS	1.2	0.9	0.9	0.9	0.7	0.6	0.6						
PZ	1.3	1.3	1.2	1.4	1.2	1.1	1.1	1.1					
BC	1.2	1.0	0.9	1.0	0.8	0.7	0.7	0.6	1.2				
LB	1.1	0.9	0.9	1.0	0.4	0.1	0.1	0.5	1.0	0.6			
HD	1.3	1.1	1.1	1.1	0.7	0.4	0.5	0.7	1.0	0.8	0.3		
BN	1.1	0.9	0.9	1.0	0.4	0.1	0.1	0.6	1.0	0.7	0.1	0.4	
**Clade B**													
	LY	ML	LM	SS	DS								
LY													
ML	0.7												
LM	1.1	0.7											
SS	0.5	0.5	0.8										
DS	0.8	0.7	1.0	0.5									
**Clade C**													
	HL	TA	TJ	PZ	BC	JS	TW	HD	SK	BN			
HL													
TA	1.4												
TJ	1.5	0.1											
PZ	1.6	0.9	1.0										
BC	1.5	0.7	0.8	0.4									
JS	1.6	0.8	0.9	0.6	0.4								
TW	1.3	0.6	0.7	0.6	0.4	0.5							
HD	0.5	1.4	1.5	1.6	1.5	1.6	1.3						
SK	0.5	1.6	1.7	1.6	1.5	1.6	1.4	0.5					
BN	0.5	1.5	1.6	1.6	1.5	1.6	1.3	0.1	0.1				

*Note*: Refer to Table 1 for the abbreviations of localities.

**TABLE 3:** The haplotype list, haplotype diversity (*h*), and nucleotide diversity (*θ*_π_) in native, released, and translocation populations based on concatenated data.

	Population	Size	Haplotype	*h*	*θ*_π_
Native					
Clade *α*	1.LY	10	41,42,43,44	0.778	0.002
	2.ML	6	45,46	0.333	0.000
	3.LM	9	37,38,39,40	0.694	0.005
	4.SS	11	14,44,54,55	0.673	0.001
	5.DS	13	13,14,15,16,17,18,19,20,21,22	0.949	0.006
Clade *β*	6.FA	9	23,24,25,26	0.806	0.002
	7.TC	9	67,68,69,70,71	0.806	0.002
	8.CK	10	6,7,8	0.511	0.002
Clade *γ*	10.TA	7	57,60,61,62,65,66	0.852	0005
	11.TJ	13	60,72,73,74	0.756	0002
	12.WS	10	74,81	0.356	0.000
	13.CS	10	9,10,11,12	0.711	0.001
Clade *δ*	14.PZ	6	4,51,52,53	0.800	0.002
	15.BC	6	2,3,4	0.733	0.001
	16.JS	11	34,35,36	0.691	0.001
	17.TW	11	75,76,77,78,79,80	0.855	0.003
Released					
Clade *β*	2.ML	4	47,48,49	0.833	0.001
Clade *γ*	14.PZ	1	50	NA	NA
	15.BC	2	1,5	1.000	0.005
Clade *δ*	9.HL	22	27,28,29,30,31,32,33	0.792	0.002
	10.TA	7	56,58,59,63,64	0.905	0.002
	11.TJ	1	56	NA	NA
Translocated					
Clade *γ*	18.LB	4	73	NA	NA
	19.HD	6	73,83,84,85,86	0.933	0.006
	21.BN	7	73,74,82	0.524	0.001
Clade *δ*	19.HD	3	88,89	0.667	0.001
	20.SK	10	88,90,91	0.733	0.001
	21.BN	2	87,88	1.000	0.001

*Note*: Underline indicated the shared haplotypes.

Among the 91 mtDNA haplotypes, only eight (4, 14, 44, 56, 60, 73, 74, 88) were shared by two or more populations (Table 3). Among eight shared haplotypes, four were shared among native populations (4, 14, 44, 60), one haplotype (56) was shared between released populations in TA and TJ, two were shared among native and translocated populations (73, 74), and only one haplotype (88) was shared among translocated populations in the east.

### 3.2 Genetic diversity and structure

**FIGURE 5:**
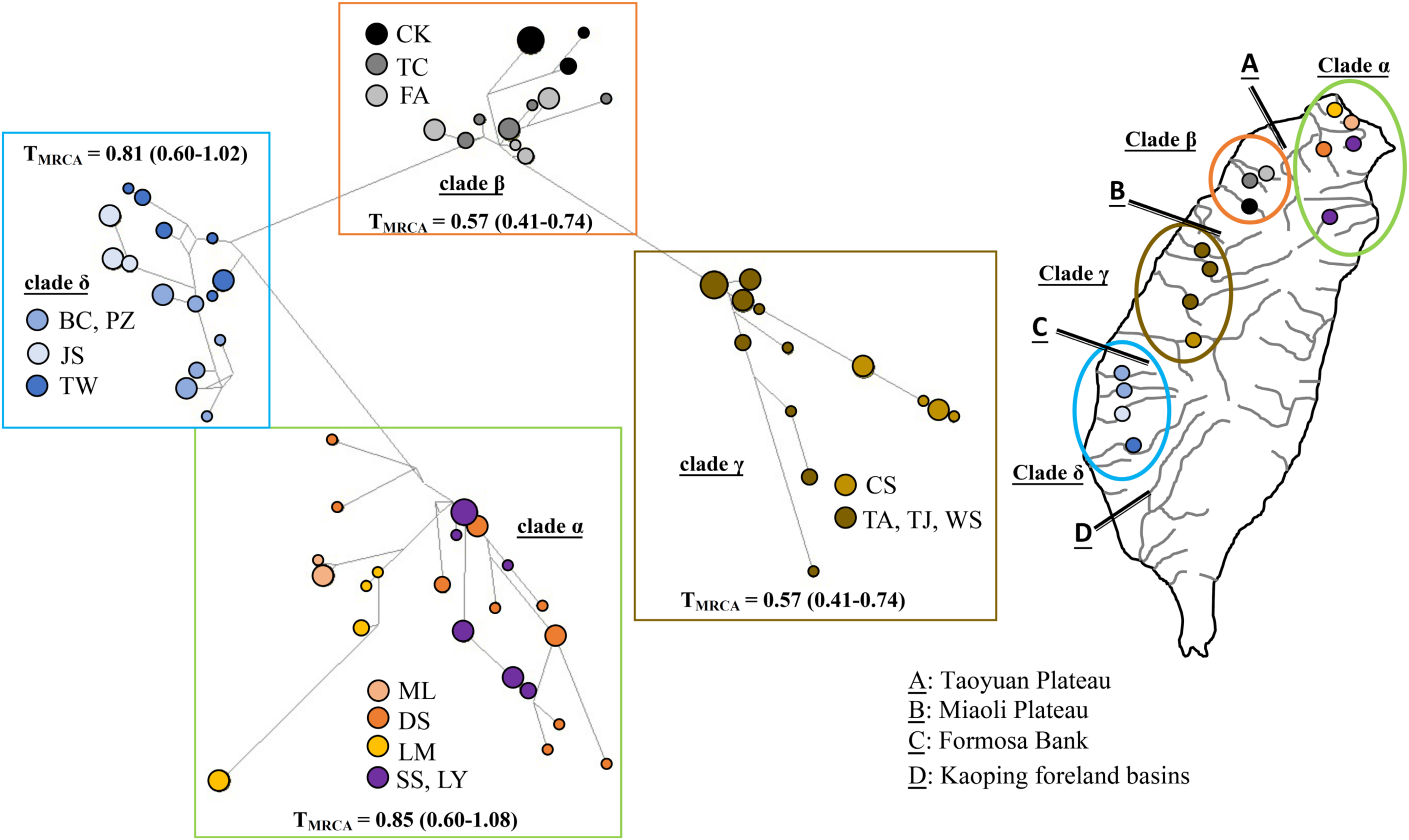
The median‐joining network‐based concatenated mtDNA sequences (1981 bps) of native *Rhinogobius candidianus* and their distribution of the clades

Our study assorted all sampling specimens as three groups, native, released, and translocated populations (Table 3). Removing the released and translocated populations, a total of 151 native specimens were re‐analyzed using concatenated data (Table 3). The haplotype network based on mtDNA data set revealed these specimens fell into four allopatric clades, *α*–*δ* (Figure 5). The clades *α*–*δ* were distributed in north, northwest, west, and southwest regions, respectively (Figure 5). The clades *β* and *γ* were clustered together as cyt *b* lineage I (Figure 2a). The results of the BEAST analysis suggested that the time of coalescence was estimated to be sometime in the late Pleistocene [*T*_MRCA_ = 0.94 mya (million years ago), 0.74–1.15]. The *T*_MRCA_ values of the four clades (*α*–*δ*; Figure 5) were 0.85 (0.60–1.08), 0.57 (0.41–0.74), 0.57 (0.41–0.74), and 0.81 (0.60–1.02), respectively. A comparison of the fixation indices *N*_ST_ and *G*_ST_ revealed that *N*_ST_ was larger than *G*_ST_ (0.841 and 0.266, respectively). This suggested a significant relationship between phylogeny and geography. The overall *F*_ST_ was 0.840. The average pairwise *F*_ST_ within north, northwest, west, and southwest regions was 0.634, 0.415, 0.538, and 0.637, respectively, and the pairwise *F*_ST_ among these four regions was 0.853, with a range from 0.794 to 0.883 (Table 4). These results showed that the population differentiations were high.

**TABLE 4:** Matrix of pairwise *F*_ST_ between native populations based on concatenated data. Refer to Table 1 for the abbreviations of localities

	ML	LM	SS	DS	FA	TC	CK	TA	TJ	WS	CS	PZ	BC	JS	TW
LY	0.843	0.694	0.692	0.530	0.869	0.884	0.879	0.801	0.898	0.930	0.901	0.882	0.881	0.897	0.820
ML		0.665	0.875	0.578	0.920	0.935	0.923	0.851	0.945	0.979	0.949	0.929	0.931	0.949	0.884
LM			0.673	0.504	0.813	0.826	0.826	0.765	0.846	0.872	0.850	0.820	0.813	0.836	0.773
SS				0.290	0.894	0.912	0.901	0.822	0.925	0.961	0.932	0.910	0.912	0.930	0.850
DS					0.759	0.776	0.776	0.706	0.794	0.823	0.805	0.776	0.765	0.783	0.709
FA						0.054*	0.592	0.667	0.804	0.858	0.800	0.858	0.855	0.874	0.793
TC							0.599	0.687	0.827	0.883	0.819	0.873	0.872	0.891	0.806
CK								0.684	0.821	0.878	0.797	0.881	0.881	0.895	0.892
TA									0.260	0.454	0.510	0.816	0.811	0.827	0.761
TJ										0.403	0.748	0.905	0.906	0.919	0.860
WS											0.853	0.938	0.941	0.952	0.895
CS												0.909	0.910	0.922	0.859
PZ													0.574	0.778	0.599
BC														0.705	0.526
JS															0.639
≤0.25	≤0.50	≤0.75	≤1.00												

*Note*: The *F*_ST_ values that are non‐significantly different from zero (*p* = .05) are marked with an asterisk (*).

*F*_st_ value decreases with shades of blue light.

**FIGURE 6:**
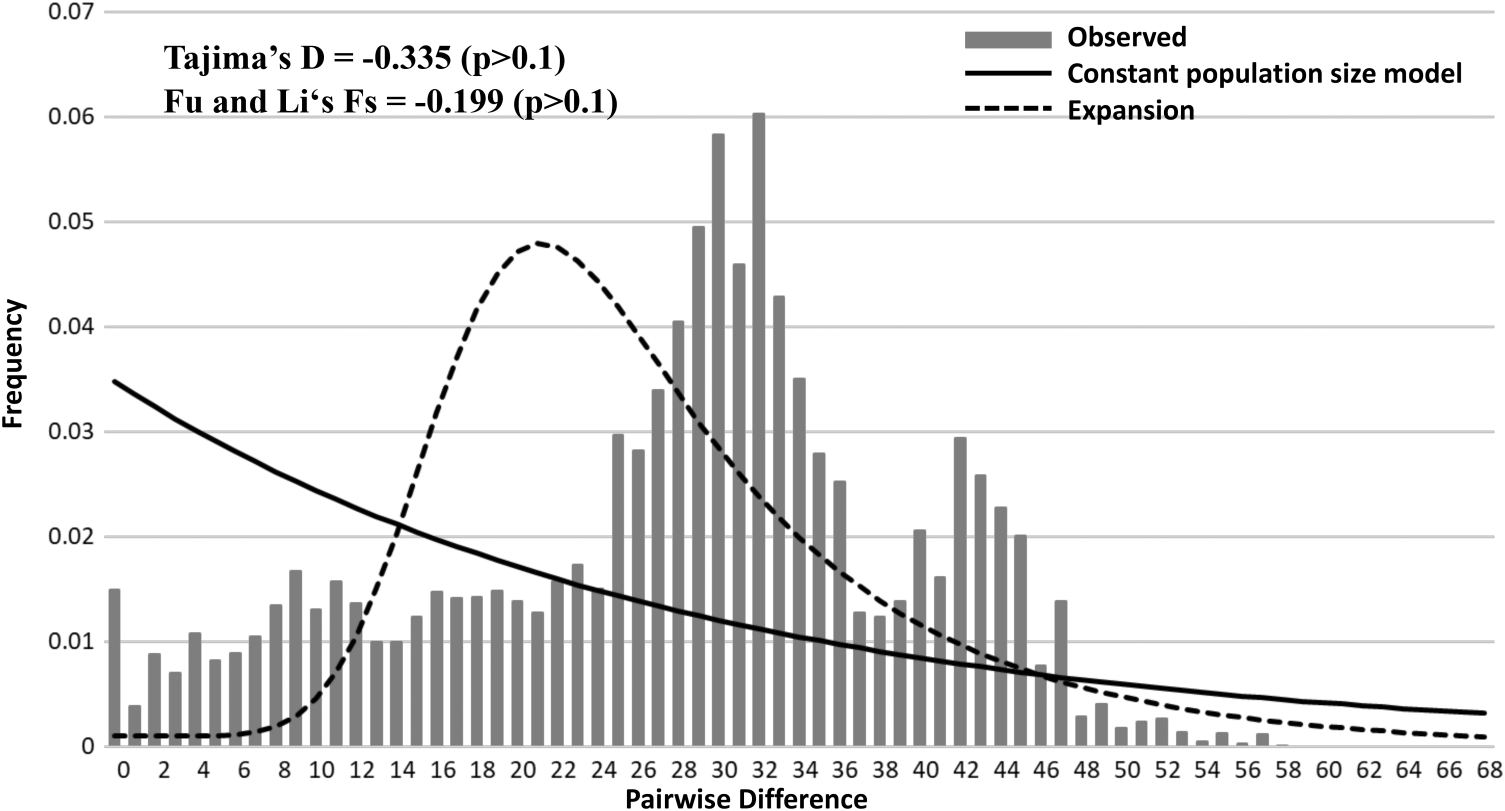
Mismatch distributions of concatenated mtDNA (1981 bps) of native and translocated *Rhinogobius candidianus*. The dotted line is the expected distribution calculated for the assumption of a demographically expanding population; black line is the expected distribution under the constant population model; gray bars indicate the observed frequencies of pairwise differences of nucleotides among haplotypes.

**FIGURE 7:**
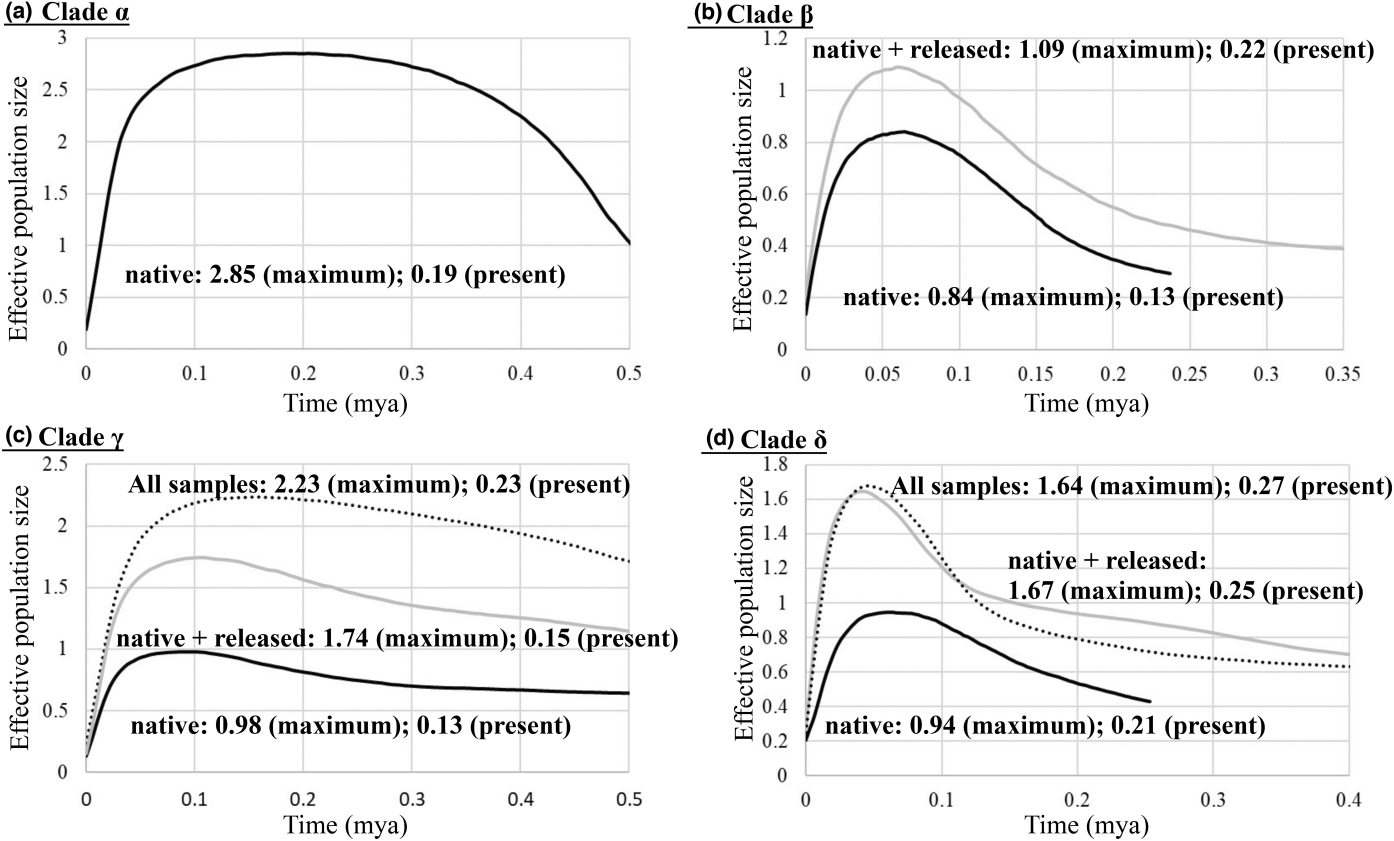
Bayesian skyline plot of changes of the effective population sizes through time for *Rhinogobius candidianus*

In all samples based on the concatenated data, Tajima's *D* and Fu's *Fs* tests, and mismatch distribution displayed non‐signature of recent demographic expansion (Figure 6). Moreover, the pattern of the mismatch distribution was multimodal, and these results supported the population differentiation (Yan et al., 2020; Yi et al., 2021). Thus, our study analyzed the demographic history in four clades based on native, native + released, and all samples (including native, released, and translocated) (Heller et al., 2013). The BSP, which simulated the fluctuations in population size over time, concluded population declined patterns in the four clades (clades *α*–*δ*, Figures 5 and 7). These results revealed that in the past, the clade *α* had higher effective population size, and the effective population sizes of other three clades were similar. However, today, the clade *δ* had higher effective population size relative to the other clades. In clade *γ*, the analyses of the BSP suggest that the effective population size in the all samples’ data set, including the translocated and released populations, revealed higher population size than that in the native + released data, excluding the translocated population (Figure 7c). In clade *δ*, the analyses of the BSP suggest that the results in the two data sets, including and excluding the translocated populations (all and native + released), revealed similar results (Figure 7d). The results in clades *γ* and *δ* showed that the translocated and released specimens did not always increase the effective population size.

**FIGURE 8:**
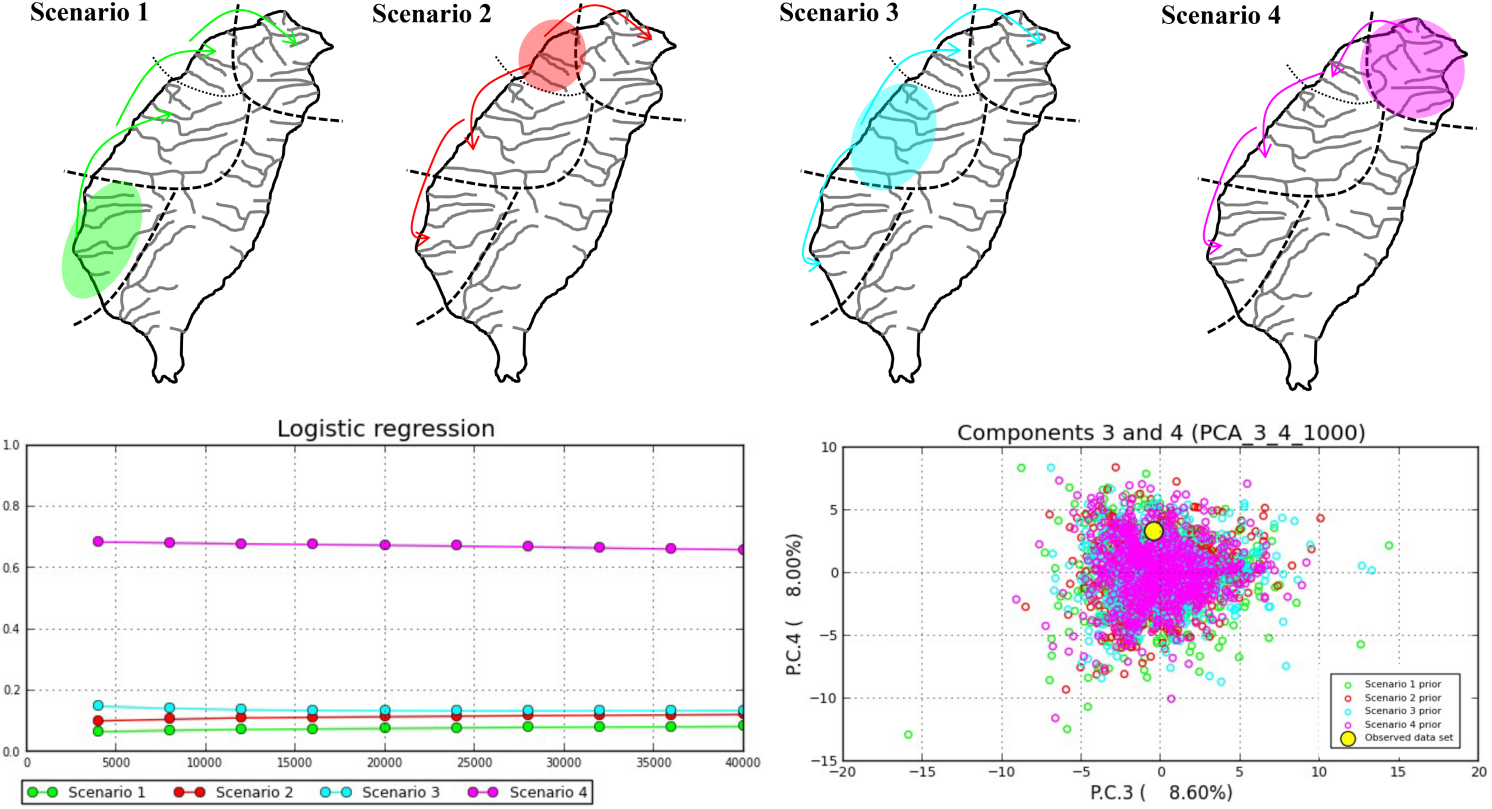
The four scenarios tested in the DIYABC analysis, the results of a logistical model comparing posterior probability of each scenario with the number of simulations used to calculate it, and the prior distribution of parameters corresponding to the four scenarios tested. Colors reflect each scenario.

The results of the S‐DIVA analysis indicated that the possible ancestral populations were distributed widely in Taiwan (all regions: 91%, and north, west, and south: 9%; data not shown). Four population history scenarios with the DIYABC program were proposed. The ancestral populations of colonized north (clade *α*), northwest (clade *β*), west (clade *γ*), and southwest (clade *δ*) Taiwan, respectively. The highest posterior probability was found for the North scenario (scenario 4; Figure 8). Its posterior probability (0.6596, 95% CI: 0.6411–0.6781) was higher than for other scenarios [scenario 1(Southwest): 0.0832, 0.0758–0.0907; scenario 2 (Northwest): 0.1219, 0.1115–0.1324; and scenario 3 (West): 0.1352, 0.1237–0.1468]. These results indicated that the ancestral populations originated in north Taiwan and then were distributed widely in Taiwan before being isolated.

## 4 DISCUSSION

### 4.1 Endemic and freshwater

Chen and Shao (1996) proposed *R. candidianus* as a new freshwater goby in Taiwan. Some studies suggest that this species is an amphidromous species and is also distributed in mainland China without evidence (e.g., Froese & Pauly, 2022; Shan et al., 2000). Moreover, there is no evidence to suggest this species is also distributed in mainland China according to a literature review that we conducted on Web of Science and NCBI (National Center for Biotechnology Information). Furthermore, the *F*_ST_ of *R. candidianus* displayed high population differentiation among native populations (Table 4); the shared haplotypes were only distributed between adjacent native populations (Table 3). These results suggested that the migrating potential of *R. candidianus* was very weak. Compared with other *Rhinogobius* species in Taiwan, the overall *F*_ST_ in *R. candidianus*, *R. delicatus*, and *R. giurinus* was 0.840, 0.711, and 0.567, respectively. *Rhinogobius delicatus* is a primary freshwater fish species endemic from Taiwan (Ju et al., 2021); and *R. giurinus* is an amphidromous species (Ju et al., 2016). In addition, the overall values of *F*_ST_ in many primary freshwater species were similar to *R. delicatus* (e.g., *F*_ST_ = 0.66 in *Aphyocypris kikuchii* see Lin et al., 2008; *F*_ST_ = 0.817 in *Semisulcospira libertina* see Chiu et al., 2017; *F*_ST_ = 0.72 in *Neocaridina davidi* see Han et al., 2019). Moreover, the shared haplotype of *R. giurinus* was distributed widely on the island (Ju et al., 2016), and that of *R. delicatus* was only shared between adjacent populations (Ju et al., 2021). Thus, our study considered that *R. candidianus* is a primary freshwater fish and Taiwan endemic species.

### 4.2 Native, translocated, and released populations

Chen and Shao (1996) discovered *R. candidianus*, and proposed this species was not distributed in east Taiwan, and in and south of the Tzengwen River (TW). However, Leander et al. (2014) found that this species was distributed in Taiwan widely and Liao et al. (2021) proposed that *R. candidianus* has been introduced to east Taiwan and became dominant. Actually, previous phylogeographic studies also found that other freshwater fish in west Taiwan were introduced to east Taiwan (*Zacco pachycephalus* see Wang et al., 1999; *Varicorhinus barbatulus* see Wang et al., 2004; *Acrossocheilus paradoxus* see Ju et al., 2018). Besides freshwater fishes, human introduction of a freshwater prawn from west to east Taiwan was also found (*Macrobrachium asperulum* see Liu et al., 2011). Ju et al. (2018) suggested that populations secondarily moved through human contact would affect the interpretation of the phylogeographic history. Thus, our study attempted to find the native distribution pattern to infer the phylogeographic history of *R. candidianus*.

Chen and Shao (1996) found that *R. candidianus* was distributed north of the Tzengwen River (population TW) and did not find *R. candidianus* in Tzengwen River (TW). However, the population TW displayed a higher number of private haplotypes relative to the other populations, and an interior position in the network (Tables 1 and 3, Figures 4 and 5). Moreover, the previous studies proposed that the rivers in and north of TW had been defined as an ichthyofauna region (e.g., Chang et al., 2016; Chiang et al., 2017; Han et al., 2019; Lin et al., 2016). Thus, our study suggests that the native range of *R. candidianus* was distributed in north, northwest, west, and south (in and north of TW) Taiwan (Figure 5). As our descriptions in the results section state (see 3.1 Native, translocated, and released populations), four populations LB, HD, SK, and BN were defined as translocated populations because they were not distributed in the native area; and some specimens in populations ML, HL, TA, TJ, PZ, and BC were defined as released populations because these specimens were distributed in the native area but showed discordant phylogenies when compared to other geographically close populations (Table 3).

**FIGURE 9:**
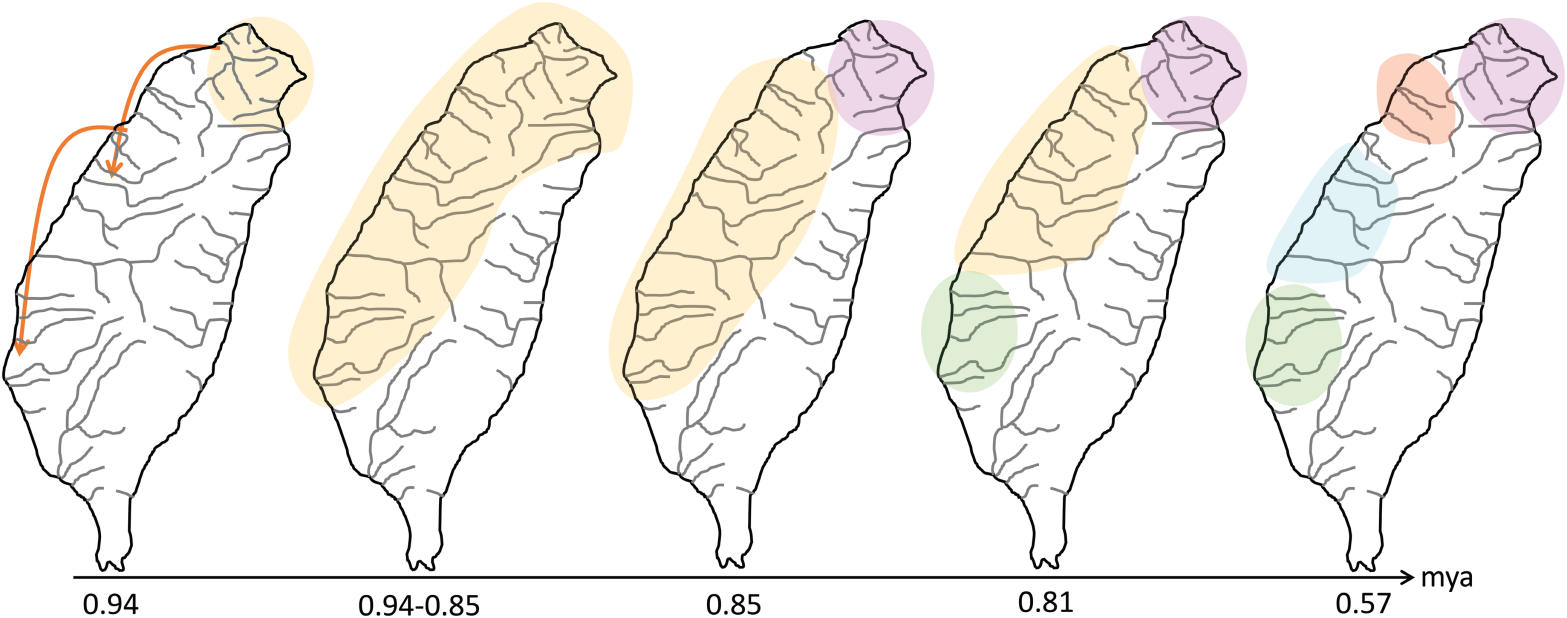
The population history of *Rhinogobius candidianus* in Taiwan

Among six released populations, population HL only included released specimens, but the other populations contained native and released specimens (Table 3). Moreover, population HL is located in the Miaoli Plateau, which separates the clades *β* and *γ* (Figures 1 and 5; see 4.3 Phylogeography of *R. candidianus*), but this population did not belong to clades *β* and *γ* and it was closer to population TW (clade *δ*, Figures 2a, 4 and 9, Table 2). The average pairwise *F*_ST_ between population HL and other populations was 0.897, with a range from 0.825 (TW) to 0.948 (WS) (data not shown). Population HL was likely one source of the translocated populations in east Taiwan considering all haplotypes of population HL were private (Figure 3c and d, Table 3); and the tree and haplotype network branches among populations HL and other native populations were longer (Figures 2 and 4). It is possible that the population HL may have undergone a bottleneck, and then expanded, but its history needs to be tested further.

### 4.3 Phylogeography of *R. candidianus*

Previous studies found that the mutation rate of mtDNA for the molecular clock calibration was variable, ranging from 0.54% (Ruber et al., 2007; Zardoya & Doadrio, 1999) to 2.20% (Van Steenberge et al., 2020). Our study used a mutation rate of 1.33% per million years to calibrate the values of the *T*_MRCA_ of each clade of *R. candidianus* based on concatenated data set according to that of *R. delicatus* in east Taiwan (Ju et al., 2021). The results of the estimated T_MRCA_ based on the concatenated data set suggested that the origin of *R. candidianus* could be traced back to late Pleistocene (*T*_MRCA_ = 0.94 mya), agreeing within previous observations (e.g., Chang et al., 2016; Ju et al., 2016, 2018). Thus, our study considered that this molecular clock is likely a proper estimate.

Taiwanese orogeny (mountain building) uplifted the longitudinal Central Range to nearly 4000 m at 2 mya (Teng, 1990). The distribution patterns of freshwater fishes and phylogeographic studies indicate that the Central Range has acted as a barrier to dispersal between the western and eastern populations of species (e.g., Chang et al., 2016; Tzeng, 1986). Thus, a pan‐island distribution of freshwater species is rare. Many freshwater species are only distributed west of the Central Range, for example, *Semisulcospira libertina* (Gastropoda), *Caridina pseudodenticulata* (Decapoda), *Pararasbora moltrechti* (Cypriniformes), and *R. rubromaculatus*; and some species are only distributed east of the Central Range, for example, *R. delicatus*, and *Hemimyzon taitungensis*. The present study revealed that the native *R. candidianus* was distributed west of the Central Range (Figures 1 and 5). This distribution pattern also suggested that *R. candidianus* colonized Taiwan after 2 mya. Besides the Central Range, the landform in the east coast of Taiwan island also broke the dispersions of *R. candidianus* from northeast to east because the land along the east coast was not exposed even during glaciations (Figure 1).

In total, 151 specimens from 16 native populations fell into four allopatric clades (Table 3, Figure 5). According to all results of the mtDNA haplotype network and ABC approaches (Figures 5 and 8), our study suggested that populations of *R. candidianus* colonized Taiwan through north Taiwan, and then southward to west and southwest Taiwan in 0.94–0.85 mya (Figures 5, 8 and 9). Moreover, the southward colonization route was broken by the Kaoping foreland basins (Figures 1 and 5). The Kaoping foreland basins, reaching 200 m depth within 3 km of shoreline, formed in 2–3 mya and could be a barrier for the southern part of the Kaoping river (Boggs et al., 1979; Chiang et al., 2013; Hsu et al., 2014; Ju et al., 2018; Lin et al., 2016; Wang et al., 2004). Previous studies have documented that the Kaoping foreland basins shaped the intraspecific structure and the distribution area of many freshwater species (e.g., Chiang et al., 2010, 2017; Han et al., 2019; Hsu et al., 2014; Lin et al., 2016).

After southward dispersions, the Taoyuan Plateau formed and the populations in north Taiwan (clade *α*) diverged (Figures 5 and 9). The Taoyuan Plateau is located in northwest Taiwan (Figure 1). Some freshwater fishes, for example, *O. evolans*, *S. argentatus*, *S. macrops*, and *H. labeo*, were only distributed in the Tamsui River, north of the Taoyuan Plateau (excluding). Chang et al. (2016) and Hsu et al. (2014) also found that the Taoyuan Plateau divided the populations of *M. brevirostris* and *S. libertina* into different lineages. The present study found that the Taoyuan Plateau isolated the *R. candidianus* populations in the Tamsui River as clade *α* (Figure 5).

The Formosa Bank is located in the south Taiwan Strait. Previous studies suggested that the Formosa Bank divided the glacial land bridge in the Taiwan Strait, but the effect of the Formosa Bank on the population dispersion within the island has not been described (e.g., Chang et al., 2016; Chiang et al., 2010; Lin et al., 2016; Oshima, 1923). However, Ju et al. (2018) proposed that during the maximum glacial period, the ridge uplifted from the Formosa Bank to the present coastline of Taiwan island. During maximum glaciation, the dispersions between the two sides of the bank through the exposed continental shelves of the island were fragmented. Thus, the populations south of the bank were isolated and diverged as clade *δ* (Figures 5 and 9). Finally, many studies suggest that the Miaoli Plateau isolated the dispersion of freshwater fishes (Chang et al., 2016; Jean et al., 2014; Lin et al., 2016). Thus, when the Miaoli Plateau emerged, the populations were isolated and began to diverge as clades *β* and *γ* (Figures 5 and 9).

### 4.4 Demography and conservation

The results of the Bayesian skyline plots showed that the population sizes of *R. candidianus* declined greatly (Figure 7). The neutral and mismatch distribution tests also supported the pattern of population decline (Figure 6). The existence of low genetic diversity, a pattern of population decline, and high population differentiation support the needs for the development of management strategies. Four clades in the haplotype network based on concatenated data (Figure 5) indicate that these four clades should be recognized as four evolutionarily significant units (ESUs) for conservation (Moritz, 1994; Ryder, 1986).

Besides in situ conservation, released and translocated populations are also methods for resource management. Our study found that the released and translocated populations, for example, HL, HD, SK, and BN, have many private haplotypes (Tables 1 and 3). The results of phylogenetic analyses also displayed that these populations were different than other native populations (Figures 2a and 4). In addition, our study found that three shared haplotypes (S03, T03, and T05) were only distributed in the east populations (translocated populations HD, SK, and BN; Figure 3c). These three haplotypes may have recently diverged from a common source in the east populations, and these three east populations likely originated from population HL (Figure 4). The population HL did not have shared haplotypes with any population (Figure 3c and d), and the phylogenetic tree and haplotype network branches among populations HL and other native populations were longer (Figures 2 and 4). Our study suggests that the population HL has a special history, relative to other native populations, that should be further explored. In total, our results suggested that all populations of *R. candidianus* in Taiwan should be recognized as six management units [MUs; four clades *α*–*δ*, released population in Miaoli Plateau (HL) and translocated populations in east Taiwan (HD, SK, and BN) (Figure 5, Table 3)] for conservation (Palsbøll et al., 2007).

In addition, our study suggested that the translocated populations need to be carefully evaluated. In clade *δ*, the analyses of BSP displayed that the results in the two data sets, including and excluding the translocated populations (all and native + released), revealed the similar effective population sizes (Figure 7d). However, in clade *γ*, the BSP results displayed that the results in the all samples, including translocated populations, had higher effective population sizes than that in native + released samples, excluding translocated populations (Figure 7c). Our study found that the nucleotide diversities of the translocated populations in the clade *γ* were higher than those in the clade *δ* (Table 3). Conclusively, if the nucleotide diversities in sources of the translocated populations were low, the work of ex situ conservation is not necessarily (Figure 7c and d). Besides, our study considered that these translocated populations in east Taiwan also need to be carefully evaluated because our study found that the body sizes are larger than those in west Taiwan, and the populations have been becoming dominant (Liao et al., 2021; our observations). Thus, it is necessary to make an evaluation about whether these introduced populations damaged other native species. The results of this research are valuable for future evaluations of the resource management and conservation of *R. candidianus*.

## 5 CONCLUSION

The phylogeography of *R. candidianus* in Taiwan island is uncertain. This study provides the evidence to confirm the populations in east Taiwan as translocated populations, and some specimens in native populations from secondarily moved by human intervention. Although our study also suggests that the population in Miaoli Plateau (HL) is a released population, its population history needs to be tested further. The effective population size of *R. candidianus* declined greatly. The population differentiations were shaped by the complex geological history of Taiwan island. These results could be useful for the evaluation of the resource management and conservation of *R. candidianus*. Besides, our study considered that translocated populations in east Taiwan need to be carefully evaluated and monitored. It is necessary to make an evaluation about whether these introduced populations damaged other native species. In future studies, we need more samples of other *Rhinogobius* species worldwide, and more molecular characters to examine the origin of *R. candidianus*.

## AUTHOR CONTRIBUTIONS

**Bin Kang:** Conceptualization (equal); software (equal); writing – original draft (equal). **Kui‐Ching Hsu:** Conceptualization (equal); methodology (equal); software (equal); writing – original draft (equal). **Jui‐Hsien Wu:** Investigation (equal); resources (equal); validation (equal). **Yuh‐Wen Chiu:** Investigation (equal); methodology (equal); resources (equal). **Hung‐Du Lin:** Conceptualization (equal); software (equal); writing – review and editing (equal). **Yu‐Min Ju:** Conceptualization (equal); funding acquisition (equal); software (equal); writing – review and editing (equal).

## CONFLICT OF INTEREST

The authors have no conflicts of interest to declare.

### OPEN RESEARCH BADGES

This article has earned Open Data, Open Materials and Preregistered Research Design badges. Data, materials and the preregistered design and analysis plan are available at GenBank (https://www.ncbi.nlm. nih.gov/) under the accession numbers OK375877–OK375931, and OK375932–OK376009.

## Data Availability

The data set, which included 55 cytochrome *b* and 78 d‐loop sequences, was submitted to GenBank (https://www.ncbi.nlm. nih.gov/) under the accession numbers OK375877–OK375931, and OK375932–OK376009.
